# Supplementary material for: Content-rich biological network constructed by mining PubMed abstracts
Source: BMC Bioinformatics. 2004 Oct 8;5:147. doi: 10.1186/1471-2105-5-147 (PMC528731; doi:10.1186/1471-2105-5-147)
Supplement: Additional File 2 — The original results of the above study (non-essential files are deleted to keep the file size under the limit set by BMC bioinformatics). [file 1471-2105-5-147-S2.bz2 › chilibotAdditionalFile2/dip50/246ID8962075E809/html/RECO_RECF.html]

 


 **RECO** and **RECF** 
  
Found 63 abstracts in PubMed, retrieved 50.  
 

 What does Google say? 
 PDF only 
| .edu only 

---

**Interactive relationship** (e.g. stimulation, inhibition, etc)

**Neutral relationship**- **RecF**  protein competes with  **RecO**  protein for RecR protein association and its addition destabilizes RecAOR filaments.  Ref: 11743007 EMBO J, 2001
- Finally, we found that interactions of  **RecF**  with  **RecO**  are lost in the presence of ATP.  Ref: 8962075 Proc Natl Acad Sci U S A, 1996
- These data suggested that  **RecO**  mediates the interactions of  **RecF**  protein with RecR and with Ssb proteins.  Ref: 8962075 Proc Natl Acad Sci U S A, 1996
- The  **recO**  gene is required for the  **RecF**  pathway of recombination and the repair of DNA daughter strand gaps in Escherichia coli.  Ref: 8538645 Mutat Res, 1996
- The  **recO**  gene product is required for  **RecF**  pathway mediated recombination and the repair of DNA damage after UV irradiation or mitomycin C exposure in Escherichia coli.  Ref: 8107098 J Mol Biol, 1994
- Since  **recF**  is epistatic with  **recO**  and recR recOR for UV resistance, recOR mutations were also tested with priA2 kan.  Ref: 8820655 Mol Microbiol, 1996
- iii  **RecF**  interacts with Ssb protein in the presence of  **RecO** .  Ref: 8962075 Proc Natl Acad Sci U S A, 1996
- This function, designated orf for  **recO** , recR and  **recF**  like function, is largely  **RecF**  pathway specific.  Ref: 1310087 Genetics, 1992
- These data suggest that one role of  **RecF**  is to stabilize the interaction of RecR with  **RecO**  in the presence of Ssb protein.  Ref: 8962075 Proc Natl Acad Sci U S A, 1996
- We observed synergistic interactions between uvrD ant the  **recF** , recN,  **recO**  and recG genes in both conjugational recombination and the repair of methylmethane sulfonate MMS induced DNA damage.  Ref: 8647383 Genetics, 1995
- No synergistic interactions were detected between helD and the  **recF** ,  **recO**  and regN genes when conjugational recombination was analyzed.  Ref: 8647383 Genetics, 1995
- We did detect synergistic interactions between helD and  **recF**   **recO**  in recombinational repair.  Ref: 8647383 Genetics, 1995
- The  **recF** ,  **recO** , and recR genes were originally identified as those affecting the  **RecF**  pathway of recombination in Escherichia coli cells.  Ref: 9537373 J Bacteriol, 1998
- We therefore assigned  **recO**  to epistatic group alpha and predict that the  **RecO**  protein acts at the same stage of recombination as the  **RecF** , RecL and RecR proteins, in a RecFLOR complex.  Ref: 10323239 Mol Gen Genet, 1999
- Biochemical interaction of the Escherichia coli  **RecF** ,  **RecO** , and RecR proteins with RecA protein and single stranded DNA binding protein.  Ref: 8483906 Proc Natl Acad Sci U S A, 1993
- Incubation of  **RecF** ,  **RecO** , RecR, and Ssb proteins resulted in the formation of  **RecF**   **RecO**  Ssb complexes ; i.e., RecR was excluded.  Ref: 8962075 Proc Natl Acad Sci U S A, 1996
- Interactions of  **RecF**  protein with  **RecO** , RecR, and single stranded DNA binding proteins reveal roles for the  **RecF**   **RecO**  RecR complex in DNA repair and recombination.  Ref: 8962075 Proc Natl Acad Sci U S A, 1996
- Preincubation of  **RecF** ,  **RecO** , and RecR proteins prior to addition of Ssb protein resulted in the formation of complexes consisting of  **RecF** ,  **RecO** , RecR, and Ssb proteins.  Ref: 8962075 Proc Natl Acad Sci U S A, 1996
- On the other hand, the presence of recA2020, a suppressor mutation for  **recF** ,  **recO** , and recR mutations, restored normal UVM in  **recF** ,  **recO** , and recR mutants.  Ref: 9537373 J Bacteriol, 1998
- It is inferred from these results that  **recF**  overexpression may reduce UV inducible sulAp expression by a mechanism that is sensitive to the ability of  **RecF**  to bind ATP and to the levels of  **RecO**  and RecR RecOR in the cell, but not to the type of DNA damage per se.  Ref: 7830722 Mol Gen Genet, 1994

**Non-interactive relationship** (e.g. studied together, co-existance, homology, etc.)

- This action of RecA does not involve the induction of the SOS response and does not require any of the recombination proteins essential for the presynaptic step of homologous recombination, RecBCD,  **RecF**  or  **RecO** .  Ref: 11069680 Mol Microbiol, 2000
- These results indicate an involvement of the  **recF** ,  **recO** , and recR genes and their products in UVM, possibly by affecting the third role of RecA in UVM.  Ref: 9537373 J Bacteriol, 1998
- **RecF** ,  **RecO** , and RecR proteins had no effect on the conversion of joint molecules to final products and only appeared to stimulate an early step in the pairing reaction.  Ref: 8483906 Proc Natl Acad Sci U S A, 1993
- Bacteriophage lambda can recombine in recBC sbcB sbcC mutant cells by using its own gene orf, the Escherichia coli  **recO** , recR, and  **recF**  genes, or both.  Ref: 7961426 J Bacteriol, 1994
- Gap repair genes  **recF** ,  **recO** , and recR were not required.  Ref: 10224240 Genetics, 1999
- Cosuppression of  **recF** , recR and  **recO**  mutations by mutant recA alleles in Escherichia coli cells.  Ref: 7687008 Mutat Res, 1993
- The defects in restart attributed to mutations in  **recF** ,  **recO**  or recR were suppressed in a recA730 lexA Def strain expressing constitutively activated RecA RecA\*.  Ref: 11929519 Mol Microbiol, 2002
- Several lines of evidence suggest that the  **recF** ,  **recO** , and recR genes function at the same step of recombination and postreplication repair.  Ref: 9537373 J Bacteriol, 1998
- The  **recO**  mutation did not affect the sensitivity of  **recF** , recR or recL cells to DNA damaging agents, increased the sensitivity of recU and addAB cells and abolished the DNA repair capacity of recH cells.  Ref: 10323239 Mol Gen Genet, 1999
- Expression of the Escherichia coli mutA mutator phenotype requires recA, recB, recC, ruvA, and ruvC gene, but not recD,  **recF** ,  **recO** , or recR genes.  Ref: 10671469 J Bacteriol, 2000
- The suppression of  **recF** , recR and  **recO**  mutations by other recA alleles was more pronounced, but none of them could exert full suppression.  Ref: 7687008 Mutat Res, 1993
- With the exception of recA718, all the mutant recA alleles examined were able to suppress the UV radiation sensitivity caused by  **recF** , recR or  **recO**  mutations, but not by the recB mutation.  Ref: 7687008 Mutat Res, 1993
- In this study, we find that other genes belonging to the RecBC and  **RecF**  sexual recombination pathways also participate in this process since mutations in recB, sbcB or  **recO**  diminish, though to different degrees, the frequency of Tn10 precise excision induced by MMC treatment or by uvrD mutants.  Ref: 10102036 Mutat Res, 1999
- The ability of recA718, recA720, recA730, recA750 and two known recA Srf alleles recA2020 and recA441 to act as suppressors of  **recF** , recR and  **recO**  mutations was examined by studying their UV radiation sensitivity in uvrA cells of Escherichia coli.  Ref: 7687008 Mutat Res, 1993
- In this work, we report that null mutations in  **recF** ,  **recO** , or recR greatly reduce UV radiation mutagenesis UVM in an assay for reversion from a Trp trpE65 to a Trp phenotypes.  Ref: 9537373 J Bacteriol, 1998
- The Escherichia coli  **RecF** ,  **RecO**  and RecR pro teins have previously been implicated in bacterial recombinational DNA repair at DNA gaps.  Ref: 11743007 EMBO J, 2001
- Some proteins, most prominently  **RecF** ,  **RecO** , and RecR, have no role in the general formulation.  Ref: 8080625 Crit Rev Microbiol, 1994
- Genetic evidence suggests that the  **RecF** ,  **RecO** , and RecR RecFOR proteins participate in a common step of DNA recombination and repair, yet the biochemical event requiring collaboration of all three proteins is unknown.  Ref: 12769856 Mol Cell, 2003
- Induction was delayed to a similar degree by mutation in  **recF** ,  **recO**  or recR.  Ref: 7862088 Mol Gen Genet, 1995
- Similar to a requirement of recJ in the suppression of  **recF** , the suppression of recR and  **recO**  mutations by mutant recA alleles also required recJ.  Ref: 7687008 Mutat Res, 1993
- Involvement of  **recF** ,  **recO** , and recR genes in UV radiation mutagenesis of Escherichia coli.  Ref: 9537373 J Bacteriol, 1998
- Although the MRF alpha 2 components are apparently not encoded by currently known homologous recombination genes such as recA,  **recF** ,  **recO** , and recR, they may fulfill an important function in assembling replisomes on arrested replication forks and products of homologous strand exchange.  Ref: 11459960 Proc Natl Acad Sci U S A, 2001
- Deletion of an open reading frame in the ninR region of lambda results in an additional requirement for  **recO** , recR and  **recF**  in order to recombine in recBC sbcB sbcC mutant cells.  Ref: 1310087 Genetics, 1992
- Addition of  **recF** , recJ, recN,  **recO** , recQ, or recR mutations had no effect on the survival of ruv recG strains.  Ref: 8132450 J Bacteriol, 1994
- a dsRNA endonuclease involved in rRNA and mRNA processing and decay, Era an essential G protein of unknown functions and  **RecO**  involved in the  **RecF**  homologous recombination pathway.  Ref: 9150881 Biochimie, 1996
- iv The recJ,  **recF** ,  **recO** , and recR gene functions are nonessential in this background.  Ref: 8349557 J Bacteriol, 1993
- The  **recO**  mutation did not affect intermolecular recombination in  **recF** , recL, recH or recU cells, but reduced by about 9 fold the incidence of intermolecular recombination in addAB cells.  Ref: 10323239 Mol Gen Genet, 1999
- In the uvrA cells, mutations in  **recF** , recR or  **recO**  genes produced a major deficiency in the repair of daughter strand gaps.  Ref: 7517004 Mutat Res, 1994
- Using plasmid derivatives that overexpress combinations of the  **recO**  and recR genes, we tested the hypothesis that high level expression of  **recO**  and recR recOR in vivo will indirectly suppress the  **recF**  mutant phenotypes mentioned above.  Ref: 8206844 J Bacteriol, 1994
- In both uvrA and uvrA recBC sbcBC backgrounds, the UV radiation sensitivity caused by  **recF** , recG, recR,  **recO** , ruvA, or ruvC mutations was partially suppressed by recA2020.  Ref: 7517004 Mutat Res, 1994
- These observations are incorporated into an expanded model for the functions of  **RecF** ,  **RecO** , and RecR proteins in the early stages of postreplication DNA repair.  Ref: 9363943 Cell, 1997
- The  **recO**  mutation did not affect intramolecular recombination in the addAB, recU,  **recF**  or recL cells, but reduced it by about 75 fold in recH cells.  Ref: 10323239 Mol Gen Genet, 1999
- Models for the roles of Orf and  **RecO** , RecR and  **RecF**  in recombination are presented.  Ref: 9335578 Genetics, 1997
- ii this pathway s requires functional recA,  **recF** ,  **recO** , and recR genes, suggesting the mechanism to be daughter strand gap repair.  Ref: 11073901 J Bacteriol, 2000
- The  **recF** ,  **recO** , and recR genes form the recFOR epistasis group for DNA repair.  Ref: 8206844 J Bacteriol, 1994
- Recombination genes  **recF** , recN,  **recO** , and recQ are not essential for dam mutant viability.  Ref: 8226701 J Bacteriol, 1993
- The similar phenotypes conferred by  **recF** , recR, and  **recO**  mutations and the observations that a number of mutant recA alleles can cosuppress these three mutations suggest that the  **recF** , recR and  **recO**  gene products may function together as a complex.  Ref: 7687008 Mutat Res, 1993
- This in turn led to the discovery of several genes, including  **recF** ,  **recO** , and recR, whose role in recombination would not otherwise have been detected.  Ref: 8080625 Crit Rev Microbiol, 1994
- Unlike the case in recBCsbcA cells, intramolecular homologous recombination of linear DNA in the recBCsbcBC cells was dependent on recA and  **recF**  as well as recN and  **recO**  gene functions, but was independent of recJ and recL gene functions.  Ref: 1557026 Mol Gen Genet, 1992
- The Escherichia coli  **RecF** ,  **RecO** , and RecR proteins were analyzed for their effect on RecA mediated pairing of single stranded circular DNA and homologous linear duplex DNA substrates.  Ref: 8483906 Proc Natl Acad Sci U S A, 1993
- To better understand the role of these two proteins in homologous recombination in the  **RecF**  pathway recBCsbcB C background, we investigated the interactions between helD, uvrD and the following  **RecF**  pathway genes  **recF** ,  **recO** , recN and ruvAB.  Ref: 8647383 Genetics, 1995
- We propose that mutation of  **recF** , recJ,  **recO** , recQ, or recR redirects recombination to events initiated by the RecBCD enzyme.  Ref: 8132450 J Bacteriol, 1994
- An increase of intracellular  **RecO** , which may be due to the increased  **recO**  gene copies or to the induction of  **RecO**  synthesis, increased the UV resistance of recA,  **recF** , and ssb cells, but did not increase the UV resistance of uvrB, uvrB  **recF** , uvrB recA and uvrB ssb cells.  Ref: 8538645 Mutat Res, 1996
- To elucidate the role of several key proteins involved in this process, we have analysed the time dependence of replication restart in strains carrying a combination of mutations in lexA, recA, polB pol II, umuDC pol V, priA, dnaC,  **recF** ,  **recO**  or recR.  Ref: 11929519 Mol Microbiol, 2002
- previously isolated mutations in B.subtilis  **recF** , recL,  **recO**  and recR, which belong to the same complementation group, all suppress the lethality of a pcrA mutation.  Ref: 12065426 EMBO J, 2002
- Mutation of  **recF** ,  **recO**  or recQ produced recombinants in which this exchange tended to be closer to the origin, though the effect observed was rather small.  Ref: 7768428 Genetics, 1995
- Altered SOS induction associated with mutations in  **recF** ,  **recO**  and recR.  Ref: 7862088 Mol Gen Genet, 1995
- **RecF** ,  **RecO**  and RecR, three of the important proteins of the  **RecF**  pathway of recombination, are also needed for repair of DNA damage due to UV irradiation.  Ref: 7862097 Mol Gen Genet, 1995
- The introduction of additional mutations in  **recF** , recJ,  **recO** , recQ, or recR is shown to increase the yield of recombinants by 6 to 20 fold via a mechanism that depends on recBC.  Ref: 8132450 J Bacteriol, 1994
- In addition, we show that  **recO**  and recR mutations delay induction of beta galactosidase activity expressed from a lexA regulated promoter following exposure of cells to UV, thus further supporting the idea that  **recF** ,  **recO**  and recR gene products are needed for induction of the SOS response.  Ref: 7862097 Mol Gen Genet, 1995
- In Escherichia coli, the recovery of replication following UV induced DNA damage is dependent upon several proteins in the  **recF**  pathway, including  **RecF** ,  **RecO** , and RecR.  Ref: 10589843 Mol Gen Genet, 1999
- The protective effect of plasmid pGam18 against radiation was completely abolished by mutations in the most  **RecF**  pathway genes  **recF** , recJ, recR,  **recO** , recQ, recN, and ruvB.  Ref: 10368783 Genetika, 1999
- Phage lambda has an analog of Escherichia coli  **recO** , recR and  **recF**  genes.  Ref: 1310087 Genetics, 1992
- **RecF**  protein by itself inhibited reactions in mixtures containing RecA and SSB, and this inhibition was overcome by the addition of  **RecO**  and RecR.  Ref: 8483906 Proc Natl Acad Sci U S A, 1993
- The results of these studies suggest that recombinational repair genes, particularly  **recF** , sbcB and  **recO**  have different roles in the induced excision of Tn10 than in recombinational mating.  Ref: 10102036 Mutat Res, 1999
- We discuss these results to explain how the  **RecF**   **RecO**  RecR complex functions as an anti Ssb factor.  Ref: 8962075 Proc Natl Acad Sci U S A, 1996
- Roles for lambda Orf and Escherichia coli  **RecO** , RecR and  **RecF**  in lambda recombination.  Ref: 9335578 Genetics, 1997
- Resistance of E. coli strains to cisplatin as determined by plating experiments decreased in the following order parent PQ30 strain, polB >  **recO** , recR,  **recF**  > recB.  Ref: 12531023 DNA Repair Amst , 2002
- No evidence for repair was obtained for the wild type or for strains with mutations in the following genes mutS, recA, recBC sbcBC, recD,  **recF** , recJ, recN,  **recO** , recR, recBC sbcBC  **recF**  uvrA, recG ruvC, ruvB, lexA3, lexA51, uvrA, nfo xth nth, polA Ts, or pcnB.  Ref: 8320213 J Bacteriol, 1993
- ii In a recBC sbcA background, in which RecE mediated recombination is active, recA, recJ, recQ, recE, recT,  **recF** ,  **recO** , and recR mutations led to their accumulation.  Ref: 12718760 BMC Mol Biol< MedlineTA>BMC Mol Biol, 1993
- Introduction of the defective lexA51 mutation lexA51 Def and or UmuD into  **recF** ,  **recO** , and recR mutants failed to restore normal UVM in the mutants.  Ref: 9537373 J Bacteriol, 1998
- dnaC suppressors of priA overcome this inviability, especially when  **RecF** ,  **RecO**  or RecR is inactivated, indicating that RdgC avoids or counters a toxic effect of these proteins.  Ref: 12554673 EMBO J, 2003
- This inhibitory effect was overcome by the addition of  **RecO**  and RecR or  **RecF** ,  **RecO** , and RecR.  Ref: 8483906 Proc Natl Acad Sci U S A, 1993
- Taken together, these results further support the notion that the  **recF** , recR and  **recO**  gene products abbreviated as RecFOR function at the same step in recombination repair, possible as a complex.  Ref: 7517004 Mutat Res, 1994
- Consistent with this interpretation was the observation that the cpeA mutant phenotype was less pronounced in  **recF** , recJ and  **recO**  mutants.  Ref: 7700238 Mol Gen Genet, 1995
- Expression of a gene s from the nin region of phage lambda partially complemented both the viability and recombination defects of the  **recF** ,  **recO** , and recR mutants and the recombination defect of ruvC but not of ruvAB or recQ mutants.  Ref: 10735883 J Bacteriol, 2000
- It was found that  **recF**  overexpression reduced sulAp expression after DNA damage by mitomycin C or nalidixic acid,  **recO**  and recR overexpression partially suppressed the reduction of UV induced sulAp expression caused by  **recF**  overexpression.  Ref: 7830722 Mol Gen Genet, 1994
- Mutations affecting the  **RecF**  pathway of recombination  **recF** , recG, recJ, recN,  **recO** , recQ, recR, ruvA, ruvC were systematically introduced into two sets of strains a uvrA and uvrA recA2020, b uvrA recBC sbcBC and uvrA recBC sbcBC recA2020.  Ref: 7517004 Mutat Res, 1994
- The phage lambda orf gene encodes a trans acting factor that suppresses Escherichia coli  **recO** , recR, and  **recF**  mutations for recombination of lambda but not of E. coli.  Ref: 7961426 J Bacteriol, 1994
- Except for the recA and lexA3 lnd mutants, recA expression was induced by mitomycin C in wild type cells and its isogenic recB, recD,  **recF** , recG, recJ, recL, recN,  **recO**  and ruv derivatives.  Ref: 8274529 Biochimie, 1993
- Finally, examples are shown for in vitro reactions involving DNA, RecA,  **RecO** ,  **RecF** , RecR, and SSB that lead to a further understanding of recombinational repair.  Ref: 11098470 Mol Biotechnol, 2000
- A  **recO**  deficient strain did not exhibit dramatic reduction in efficiency of IRR, implying that  **RecF**  and RecR proteins maintain function during this mechanism in the absence of functional  **RecO** .  Ref: 8921869 Gene, 1996
- Mutation of  **recF** , recJ,  **recO** , recQ, or recR improves Hfr recombination in resolvase deficient ruv recG strains of Escherichia coli.  Ref: 8132450 J Bacteriol, 1994
- However, this clone does not complement a  **recO**  mutation for conjugational recombination or  **recO** , recR, or  **recF**  mutations for repair of UV induced DNA damage.  Ref: 7961426 J Bacteriol, 1994
- The products of the  **recF** ,  **recO** , and recR genes are thought to interact and assist RecA in the utilization of single stranded DNA precomplexed with single stranded DNA binding protein Ssb during synapsis.  Ref: 8962075 Proc Natl Acad Sci U S A, 1996
- The stimulatory effect of  **RecF** ,  **RecO** , and RecR was not seen without SSB or when SSB was added after RecA.  Ref: 8483906 Proc Natl Acad Sci U S A, 1993
- We compared the recombination proficiencies of recB1080,  **recO** , recR, and  **recF**  single mutants and recB1080  **recO** , recB1080 recR, and recB1080  **recF**  double mutants.  Ref: 12618388 Genetics, 2003
- This pathway operating on conjugational recombination requires the recA,  **recF** , recJ, recN,  **recO** , recQ, recR, ruvA, ruvB and ruvC genes.  Ref: 1310087 Genetics, 1992
- These data suggest that  **RecO**  and RecR, and possibly  **RecF** , help RecA overcome inhibition by SSB and utilize SSB single stranded DNA complexes as substrates.  Ref: 8483906 Proc Natl Acad Sci U S A, 1993
- Similarly,  **recF** ,  **recO**  or recR mutations suppress the lethality of the Escherichia coli rep uvrD double mutant.  Ref: 12065426 EMBO J, 2002
- To elucidate the mechanism that underlies this process, we examined protein protein interactions between RecA,  **RecF** ,  **RecO** , RecR, and SSB, and characterized the structure and activity of the ssDNA complexes formed with different combinations of these proteins.  Ref: 7962001 J Biol Chem, 1994
- In a wild type, lexA background, mutations in  **recF** ,  **recO**  or recR block both pathways.  Ref: 11929519 Mol Microbiol, 2002
- Bacteriophage lambda lacking its Red recombination functions requires either its own gene product, Orf, or the product of Escherichia coli s  **recO** , recR and  **recF**  genes RecORF for efficient recombination in recBC sbcB sbcC mutant cells the  **RecF**  pathway.  Ref: 9335578 Genetics, 1997
- This function is countered by the  **RecF**   **RecO**  competition for association with the RecR protein.  Ref: 11743007 EMBO J, 2001
- In this genetic background, mutations in  **recF** ,  **recO** , and recR had large effects on both cell viability and on recombination.  Ref: 10735883 J Bacteriol, 2000
- Using immunoprecipitation, size exclusion chromatography, and Ssb protein affinity chromatography in the absence of any nucleotide cofactors, we have obtained the following results i  **RecF**  interacts with  **RecO** , ii  **RecF**  interacts with RecR in the presence of  **RecO**  to form a complex consisting of  **RecF** ,  **RecO** , and RecR  **RecF**   **RecO**  RecR.  Ref: 8962075 Proc Natl Acad Sci U S A, 1996
